# Cannabidiol Ameliorates Monocrotaline-Induced Pulmonary Hypertension in Rats

**DOI:** 10.3390/ijms21197077

**Published:** 2020-09-25

**Authors:** Olga Sadowska, Marta Baranowska-Kuczko, Anna Gromotowicz-Popławska, Michał Biernacki, Aleksandra Kicman, Barbara Malinowska, Irena Kasacka, Anna Krzyżewska, Hanna Kozłowska

**Affiliations:** 1 Department of Experimental Physiology and Pathophysiology, Medical University of Białystok, 15-222 Białystok, Poland; olga.sadowska@umb.edu.pl (O.S.); mabar@umb.edu.pl (M.B.-K.); olakicman@gmail.com (A.K.); bmalin@umb.edu.pl (B.M.); anna_krzyzewska.96@wp.pl (A.K.); 2 Department of Clinical Pharmacy, Medical University of Białystok, 15-222 Białystok, Poland; 3 Department of Biopharmacy, Medical University of Białystok, 15-222 Białystok, Poland; anna.gromotowicz@umb.edu.pl; 4 Department of Analytical Chemistry, Medical University of Białystok, 15-222 Białystok, Poland; michal.biernacki@umb.edu.pl; 5 Department of Histology and Cytophysiology, Medical University of Bialystok, 15-222 Bialystok, Poland; kasacka@umb.edu.pl

**Keywords:** cannabidiol, pulmonary hypertension, isolated vessels, PAI-1, t-PA, endocannabinoids, monocrotaline

## Abstract

Cannabidiol (CBD) is known for its vasorelaxant (including in the human pulmonary artery), anti-proliferative and anti-inflammatory properties. The aim of our study was to examine the potential preventive effect of chronic CBD administration (10 mg/kg/day for three weeks) on monocrotaline (MCT)-induced pulmonary hypertension (PH) rats. PH was connected with elevation of right ventricular systolic pressure; right ventricle hypertrophy; lung edema; pulmonary artery remodeling; enhancement of the vasoconstrictor and decreasing vasodilatory responses; increases in plasma concentrations of tissue plasminogen activator, plasminogen activator inhibitor type 1 and leukocyte count; and a decrease in blood oxygen saturation. CBD improved all abovementioned changes induced by PH except right ventricle hypertrophy and lung edema. In addition, CBD increased lung levels of some endocannabinoids (anandamide, *N*-arachidonoyl glycine, linolenoyl ethanolamide, palmitoleoyl ethanolamide and eicosapentaenoyl ethanolamide but not 2-arachidonoylglycerol). CBD did not affect the cardiopulmonary system of control rats or other parameters of blood morphology in PH. Our data suggest that CBD ameliorates MCT-induced PH in rats by improving endothelial efficiency and function, normalization of hemostatic alterations and reduction of enhanced leukocyte count determined in PH. In conclusion, CBD may be a safe, promising therapeutic or adjuvant therapy agent for the treatment of human pulmonary artery hypertension.

## 1. Introduction

Pulmonary artery hypertension (PAH) is a complex, chronic and multi-factorial disease which can lead, among other complications, to an increase in right ventricle pressure (RVP), resulting in progressive right heart failure and premature death. PAH is associated with endothelial dysfunction, excessive constriction of pulmonary arteries (PAs), vascular remodeling (smooth muscle cell proliferation and hypertrophy), infiltration of inflammatory cells into the lung and thrombosis. Unfortunately, PAH remains an incurable devastating disease that urgently needs new and better therapeutic interventions [1].

Cannabidiol (CBD) is a non-psychoactive compound of *Cannabis sativa* var. *indica*. It has a broad therapeutic potential resulting from its anti-inflammatory, antioxidant, anticonvulsant, antipsychotic, anxiolytic and antiproliferative properties. It is approved by the US Food and Drug Administration for the treatment of resistant epilepsy, and it is indicated in the therapy of neuropathic pain in multiple sclerosis and other diseases [2,3].

CBD causes relaxation of human mesenteric arteries [4] and rat aorta [5] and femoral [6] and mesenteric arteries under normo- and hypertensive conditions [7]. Inhalation is the most common route of recreational cannabis consumption in humans [8]. However, in contrast to systemic circulation, which is relatively well-examined, there has been only one publication so far concerning the pulmonary arterial bed. In that study, we showed that CBD fully relaxed isolated human pulmonary arteries [7]. The similarly strong relaxation of human and rat pulmonary arteries (hPAs, rPAs) caused by other exo- (abnormal-cannabidiol) and endocannabinoids (anandamide, virodhamine, 2-arachidonyl glycerol and l-alpha-lysophosphatidylinositol [9,10,11,12,13,14]) led to suggestions about the potential therapeutic significance of cannabinoids in PAH treatment [15]. In addition, CBD can modify endocannabinoid levels as a result of inhibition of fatty acid amide hydrolase (FAAH), an enzyme responsible for degradation of anandamide or other endocannabinoids, and/or its interaction with the anandamide membrane transporter. Such model-dependent modification of cardiac and plasma endocannabinoid levels after chronic CBD administration has been demonstrated in experimental hypertension [16].

Other beneficial effects of CBD in lung diseases include inhibition of (1) inflammation and/or (2) remodeling processes, demonstrated in mice acute lung injury [17] and/or experimental allergic asthma [18] and its anti-proliferative properties, i.e., in lung cancer [19,20]. Moreover, CBD inhibited platelet aggregation ex vivo induced by collagen in rats [21] and by adenosine or epinephrine in humans [22] and decreased expression and secretion of plasminogen activator inhibitor-1 (PAI-1) in human lung carcinoma [23].

Despite the favorable therapeutic properties of CBD, the long-term consequences of its treatment on the cardiopulmonary system and hemostasis under physiological and pathophysiological conditions are unknown. Thus, the aim of our study was to examine the effects of chronic preventive treatment with CBD in rat experimental pulmonary hypertension (PH). Note that the terms “PAH” and “PH” are reserved for the human and experimental conditions, respectively [24,25,26,27]. To evaluate the efficacy of CBD in PH, right ventricular systolic pressure (RVSP), relaxant-constrictor responses and remodeling of isolated PAs, as well as parameters of hemostasis and levels of endocannabinoids and/or endocannabinoid-like compounds in rat monocrotaline (MCT)-induced PH were measured.

## 2. Results

### 2.1. Effect of PH and Chronic Administration of CBD on RVSP, the Fulton Index, Lung/Weight Ratio and Blood Oxygen Saturation

**Figure 1:**
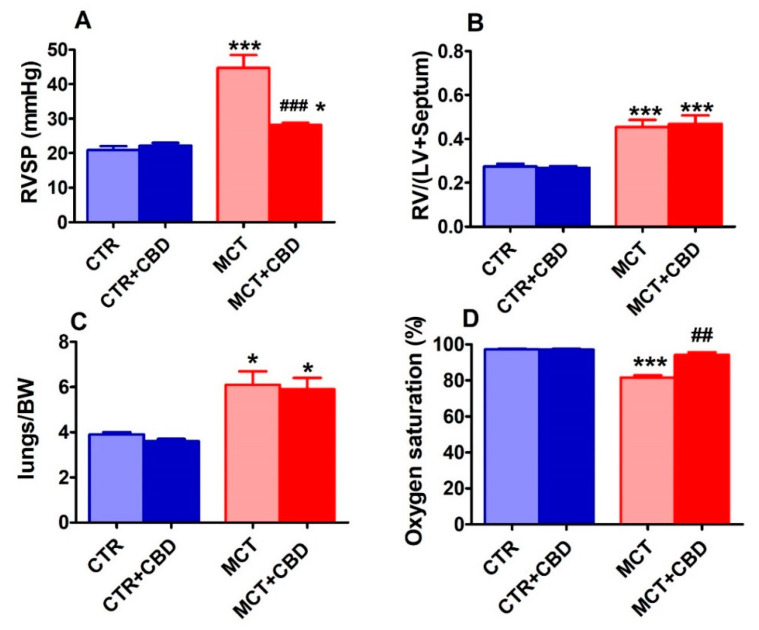
The effect of chronic administration of cannabidiol (CBD) on the right ventricular systolic pressure (RVSP) (**A**), the RV hypertrophy (ratio of the weight right ventricle to the left ventricle plus septum (RV/(LV+S)) (**B**), lung weight to body weight (BW) ratio (**C**) and oxygen saturation (**D**) in control (CTR) and monocrotaline (MCT)-induced pulmonary hypertension. CBD 10 mg/kg or its vehicle was injected intraperitoneally every 24 h for 21 days. Data are presented as mean ± SEM (*n* = 6–14 per group); * *p* < 0.05, ^##^ *p* < 0.01, ***, ^###^ *p* < 0.001, compared to the respective control groups (* CTR or ^#^ MCT).

In control (CTR) rats the RVSP, Fulton index (the right ventricle weight to left ventricle plus septum (RV/LV+S) ratio used to assess right ventricular (RV) hypertrophy), the lung weight to body weight (BW) ratio and blood oxygen saturation were equal 20.9 ± 1.1 mmHg, *n* = 14; 0.30 ± 0.01, *n* = 14; 3.9 ± 0.1, *n* = 6 and 97.1% ± 0.4%, *n* = 14, respectively. A single injection of MCT increased RVSP by about 115% (*p* < 0.001, Figure 1A), Fulton index by about 55% (*p* < 0.001, Figure 1B) and the lung weight to BW ratio by about 55% (*p* < 0.05, Figure 1C) and decreased oxygen saturation by about 15% (*p* < 0.001; Figure 1D) when compared to CTR.

CBD reduced the MCT-induced increase in RVSP by almost 80% (Figure 1A) and completely improved oxygen saturation (Figure 1D) but did not modify the MCT-stimulated changes in ratios of RV/(LV+S) (Figure 1B) or the lung weight to BW ratio (Figure 1C). In CTR animals CBD did not modify RVSP, Fulton index, the lung weight to BW ratio or oxygen saturation (Figure 1).

### 2.2. Effect of PH and Chronic Administration of CBD on Pulmonary Artery Remodeling

**Figure 2:**
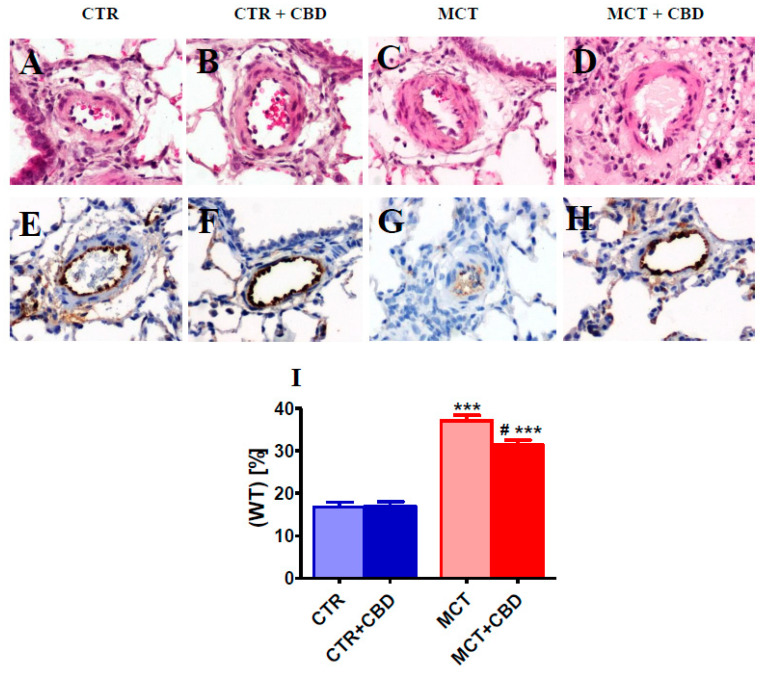
The effect of chronic administration of cannabidiol (CBD) on pulmonary vascular remodeling (**A**–**D**,**I**) and von Willebrand factor immunoreactivity (**E**–**H**) in the rat pulmonary small arteries. Representative images of occluded vessels (only found in MCT-induced pulmonary hypertension (**G**)) and percentage of medial wall thickness (WT.%) (**I**) are presented as mean ± SEM in *n* = 8 per group. CBD 10 mg/kg or its vehicle was injected intraperitoneally every 24 h for 21 days. Representative photomicrographs magnification 200×. CTR—control, MCT—monocrotaline. ^#^ *p* < 0.05, *** *p* < 0.001 compared to the respective control groups (* CTR or ^#^ MCT).

MCT-treated rats developed exaggerated elevations in pulmonary vascular remodeling. Thus, the percentage wall thickness (WT.%) of pulmonary arteries measured to evaluate pulmonary vascular remodeling increased by about 120% in MCT group compared to CTR (*p* < 0.001). CBD administration diminished the increase in WT.% observed in MCT-treated rats by about 30% (*p* < 0.05 vs. MCT) (Figure 2A,C,D,I). CBD had no effect on the pulmonary artery medial wall thickness in the CTR group (Figure 2B,I).

### 2.3. Effect of PH and Chronic Administration of CBD on Pulmonary Artery Vasoreactivity

**Figure 3:**
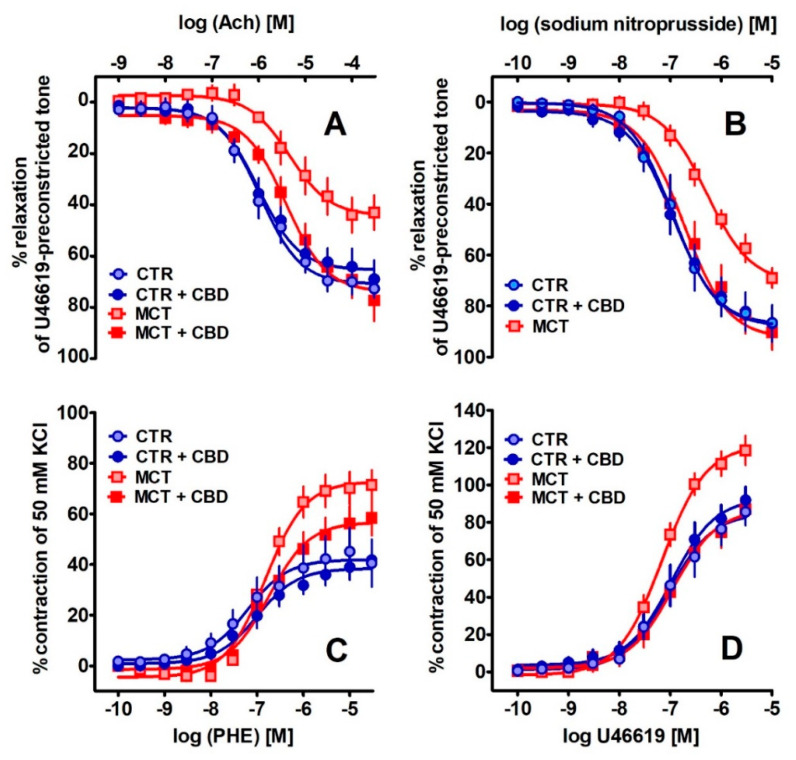
The effect of chronic administration of cannabidiol (CBD) on the vasorelaxant responses to acetylcholine (Ach) (**A**) and sodium nitroprusside (SNP) (**B**) and vasoconstrictor effects of phenylephrine (PHE) (**C**) and thromboxane A_2_ analogue (U46619) (**D**) in the pulmonary arteries of control (CTR) and monocrotaline (MCT)-induced pulmonary hypertension. CBD 10 mg/kg or its vehicle was injected intraperitoneally every 24 h for 21 days. Vasodilator and contractile responses are shown as a percentage of the isometric contraction induced by U46619 (**A**,**B**), and of the reference response to KCl (**C**,**D**), respectively. Data are presented as mean ± SEM (*n* = 7–8) tissues for each curve. In a few cases, SEM is smaller than or equal to the size of the symbols. See Table 1 for statistical analysis.

Acetylcholine (Ach, Figure 3A) and sodium nitroprusside (SNP, Figure 3B) produced a concentration-dependent relaxation of the isolated rat pulmonary arteries (rPAs) pre-constricted with the thromboxane A2 analogue U46619. The concentration-response curves (CRCs) for Ach and SNP were shifted to the right by a factor of five and the potency and efficacy were decreased in arteries of MCT-treated rats. Chronic administration of CBD to MCT-treated rats completely restored (in comparison to values of CTR animals) the efficacy of both vasorelaxants and potency of SNP, but not Ach. CBD did not modify CRCs in the CTR group (for the pEC_50_ and E_max_ values, see Table 1 and Figure 3A,B).

The α_1_-adrenoceptor agonists phenylephrine and U46619 induced a concentration-dependent contraction of the rPAs of CTR and MCT rats (Figure 3C,D). In PH only the efficacy (but not the potency) of both vasoconstrictors increased by about 75% and 40%, respectively. Chronic administration of CBD diminished or tended to reduce the efficacy of U46619 and phenylephrine, respectively, in MCT-treated rats and did not change the potencies of their vascular responses. CBD did not affect vasocontractility in the CTR group (Table 1 and Figure 3C,D).

### 2.4. Effect of PH and Chronic Administration of CBD on the Lung Levels of Endocannabinoids and Endocannabinoid-Related Lipids

**Figure 4:**
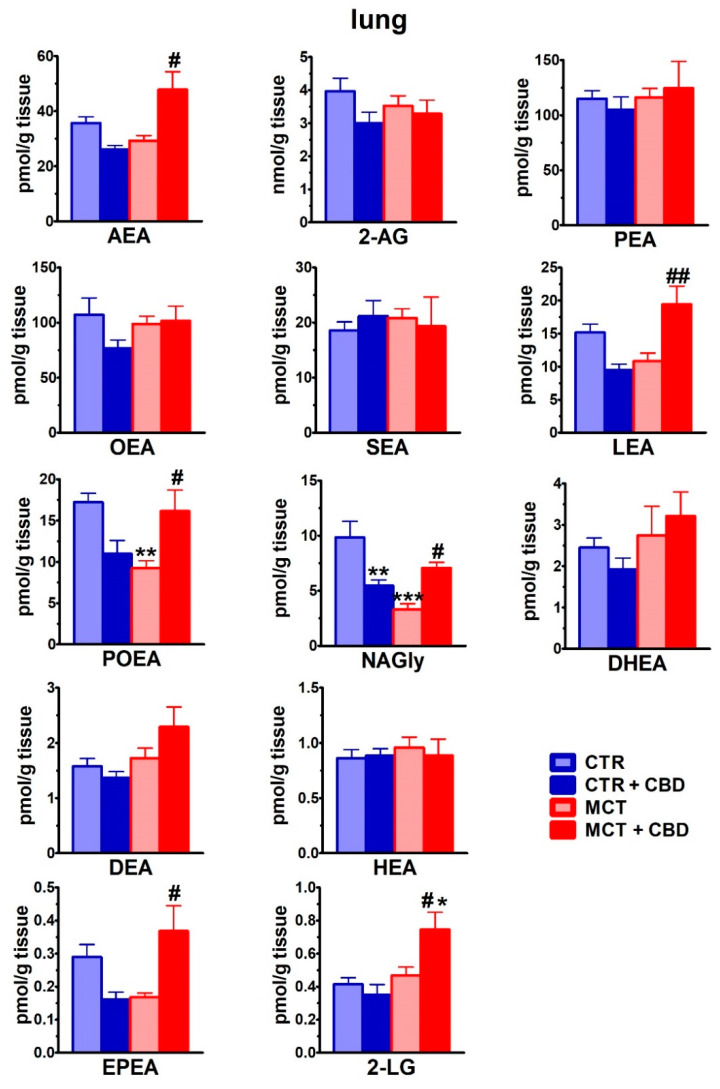
The effect of chronic administration of cannabidiol (CBD) on endocannabinoid concentrations in lungs isolated from the control (CTR) and monocrotaline (MCT)-induced pulmonary hypertension. CBD 10 mg/kg or its vehicle was injected intraperitoneally every 24 h for 21 days. Data are presented as mean ± SEM (*n* = 6 per group); *^,#^ *p* < 0.05, **^,##^ *p* < 0.01, ***p < 0.001 compared to the respective control groups (* CTR, ^#^ MCT). AEA—Anandamide; 2-AG—2-arachidonoylglycerol; PEA—palmitoyl ethanolamide, OEA—oleoyl ethanolamide; SEA—stearoyl ethanolamide; LEA—linolenoyl ethanolamide; POEA—palmitoleoyl ethanolamide; NAGly—*N*-arachidonoyl glycine; DHEA—docosahexaenoyl ethanolamide; DEA—docosatetraenoyl ethanolamide; HEA—homo-γ-linolenyl ethanolamide; 2-LG—linoleoylglycerol; EPEA—eicosapentaenoyl ethanolamide.

In CTR lungs of rats, 2-arachidonoylglycerol (2-AG) had the highest concentration among the endocannabinoids (Figure 4). The lung concentration of the well-known endocannabinoid anandamide (AEA) was relatively low (~100 times lower than that of 2-AG). Although the concentrations of palmitoyl ethanolamide (PEA) and oleoyl ethanolamide (OEA) were higher than those of AEA, they were still lower than 2-AG concentrations by ~40-fold. Lung concentrations of other endocannabinoids were lower than AEA levels. MCT decreased levels of palmitoleoyl ethanolamide (POEA) and *N*-arachidonoyl glycine (NAGly) in comparison to CTR by ~45% and ~65%, respectively. Moreover, it tended to reduce levels of eicosapentenoyl ethanolamide (EPEA), linolenoyl ethanolamide (LEA) and AEA by about 40%, 30% and 20%, respectively. Contrastingly, chronic CBD administration increased levels of AEA (~65%), linoleoylglycerol (2-LG ~60%), LEA (~80%), POEA (~75%), EPEA (~120%) and NAGly (~115%) in lungs of MCT-treated rats. In CTR rats, CBD decreased the NAGly level only by about 45% (Figure 4).

### 2.5. Effect of PH and Chronic Administration of CBD on Hemostatic Parameters and Blood Morphology

**Figure 5:**
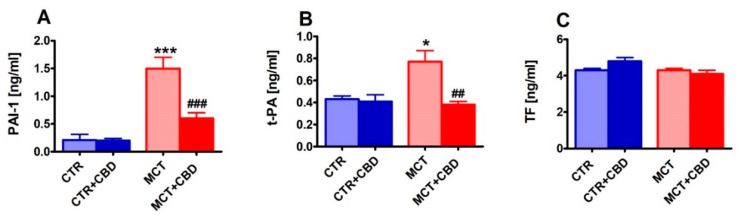
The effect of chronic administration of cannabidiol (CBD) on plasma hemostasis parameters (plasminogen activator inhibitor type 1, PAI-1 (**A**); tissue plasminogen activator t-PA (**B**); tissue factor, TF (**C**)) in control (CTR) and monocrotaline (MCT)-induced pulmonary hypertension. CBD 10 mg/kg or its vehicle were injected intraperitoneally every 24 h for 21 days. Data are presented as mean ± SEM, (*n* = 6–8 per group); * *p* < 0.05, ^##^ *p* < 0.01, ***^, ###^ *p* < 0.001 compared to the respective group (* CTR, ^#^ MCT group).

Plasma PAI-1, tissue plasminogen activator (t-PA) levels and white blood cell (WBC) count increased in MCT-treated rats by about seven times, to 80% and 110% respectively, in comparison to CTR. The MCT-induced elevation of the above parameters was markedly reduced by CBD by about four times, completely and by about 65%, respectively. No changes were observed in blood tissue factor (TF) level or in platelet adhesion to collagen, bleeding time (BT) or other parameters of blood morphology in any group after MCT or CBD treatment (Table 2, Figure 5).

## 3. Discussion

The aim of our study was to examine the potential preventive effect of chronic CBD administration (10 mg/kg/day for three weeks) on changes in pulmonary hemodynamic characteristics, vascular function and morphology, chosen hemostatic and blood morphology parameters and lung endocannabinoid levels induced in an experimental model of PH in rats. We applied the model of MCT-stimulated PH because it shows a selective toxic effect on pulmonary vessels without an effect on systemic vessels and has consistent pathological findings of PAH in humans and it serves as a preclinical model of PAH to test potential therapeutic agents [24,26].

Chronic CBD 10 mg/kg/day administration was effective in the modification of cardiac and plasma endocannabinoid levels in two models of systemic hypertension [16] and exerted beneficial cardiovascular effects in diabetic and septic rats, in mice with diabetic- and doxorubicin-induced cardiomyopathy and experimental autoimmune myocarditis (see [16]). We decided to examine the protective but not therapeutic effect of CBD, because three weeks after MCT administration rats exhibited a higher mortality rate [25].

### 3.1. Changes Related to PH

Three weeks after MCT injection we observed increases in the following, parameters of the pulmonary (but not systemic heart rate (HR) and systolic blood pressure (SBP)) circulation, which are characteristic for PH—(1) RVSP by about 115%, (2) RV hypertrophy expressed as Fulton index (RV/LV and septum ratio) by about 55% and (3) the adjusted weight of the lungs (by about 55%) suggesting lung edema [28]. The above hemodynamic alternations in MCT-treated rats could result from morphometric and vascular functional changes, such as endothelial dysfunction; vascular remodeling, as evidenced by media hypertrophy (similarly to [24,28]); and/or changes in vascular responsiveness, i.e., excessive PA constriction to thromboxane A_2_ analogue (U46619) and phenylephrine and attenuation of vasodilatory effects of Ach and SNP, as has been also demonstrated by Christou et al. [29]. The latter could result (among other potential causes) from the altered expression and function of receptors for the respective agonists in the pulmonary circulation [30]. The consequence of MCT-induced PH was relatively lower arterial oxygen saturation. PAI-1 inhibits t-PA by rapid formation of an enzyme inhibitor complex, leading to a prothrombotic phenotype of the endothelium [31]. Moreover, PAI-1 has been demonstrated to induce pulmonary vascular remodeling [32]. Similar elevations of PAI-1 and t-PA levels were observed previously in MCT-induced PH rats [32,33]. In contrast to increases in plasma t-PA and PAI-1 concentrations MCT did not modify the plasma level of TFs, platelet adhesion to collagen or bleeding time. Importantly, routine blood testing revealed more than a twofold increase in leukocyte count. This may indicate an inflammatory process, which has been shown to play an important role in PH pathogenesis [24]. However, the exact significance of these latter changes requires detailed study.

We are the first researchers who simultaneously determined 13 endocannabinoids and endocannabinoid-related lipids in the lungs (levels of OEA, SEA, HEA, DEA, 2-LG, DHEA, POEA and EPEA have never been determined in the lung). We confirmed that similarly to the lungs of rabbits [34], rats [35], mice [36] and rat hearts [16], the lungs’ 2-AG concentration was higher (by about 100 times) than the concentration of the well-known endocannabinoid AEA. Moreover, we noticed a similar ordering of endocannabinoid concentrations in rat lungs (the current study) and heart [16]: 2-AG > PEA ≈ OEA > AEA > LEA ≈ SEA ≈ POEA ≈ NAGly > DHEA ≈ DEA ≈ HEA ≈ 2-LG. Changes in endocannabinoid levels appear to be dependent on the tissue and the model of hypertension. Thus, cardiac concentrations of AEA, 2-AG and some other endocannabinoids decreased in primary arterial hypertension (spontaneously hypertensive, SHR), but increased in secondary arterial hypertension (deoxycorticosterone (DOCA-salt)) [16]. In contrast, only POEA and NAGly concentrations decreased in the lungs of PH rats.

To date, the role of these endocannabinoids and endocannabinoid-related lipids in the physiology and pathophysiology of the cardiopulmonary system, including PH, is still unknown. As mentioned in the introduction, a few of them regulate pulmonary vascular tone [9,10,11,13]. However, in the isolated ventilated and buffer-perfused lungs of mice [37] and rabbits [34], AEA (but not 2-AG) has been shown to increase the perfusion pressure (reflecting a vasopressor response) by means of its vasoactive products.

### 3.2. Influence of CBD on the MCT-Induced PH

Chronic administration of CBD (10 mg/kg for three weeks) ameliorated MCT-induced PH in rats. Thus, it reduced by almost by 80% and completely returned to the control value two important changes typical for PH that were stimulated by MCT, namely, (1) elevation in RVSP and (2) decreased blood oxygen saturation, respectively. Interestingly, chronic administration of the same dose of CBD failed to modify blood pressure (BP) and HR in rats with primary (SHR) and secondary (DOCA-salt) hypertension as well as in their normotensive controls [16]. We confirmed the lack of a CBD influence on the latter cardiovascular parameters of systemic circulation. Thus, it seems that CBD is sufficient to reduce pulmonary but not systemic BP.

How can we explain the beneficial CBD effects in PH? We suggest that the favorable effects of CBD in MCT-induced PH appear to be related mainly to the reduction of pulmonary vascular resistance by this compound, which could result from the reasons listed below.

Firstly, CBD diminished PA hypertrophy by approximately 30%. The potential anti-hypertrophic impact of CBD was demonstrated in previous experiments in which CBD decreased remodeling processes in the model of allergic asthma [18] and inhibited the proliferation and migration of human umbilical artery smooth muscle cells (HUASMCs) [38].

Secondly, CBD attenuated the vasoconstriction of PAs induced by thromboxane A_2_ analogue (significantly) and phenylephrine (tendency) and improved endothelial-dependent (Ach) and endothelial-independent (SNP) relaxation. Similar chronic administration of CBD-induced beneficial functional changes regarding systemic vascular responsiveness have been described in Zucker diabetic fatty rats [39], in hypertensive DOCA-salt and SHR rats [40] and in human brachial artery (improvement of endothelial function and arterial stiffness) [41]. The direct vasorelaxant properties of acute CBD administration in isolated arteries [4,7], including human pulmonary arteries [7], was described in the introduction. In experiments conducted in vivo, an acute CBD injection reduced diastolic but not systolic BP in pithed rats (dependent on peripheral resistance and cardiac work, respectively) [42], as well as the stress-induced increase in SBP in patients [41].

Thirdly, CBD increased lung concentrations of endocannabinoids (AEA, 2-LG, LEA, POEA, EPEA and NAGly). AEA and NAGly have been demonstrated to possess potential vasodilatory properties, as has AEA in hPAs and rPAs [10] and NAGly in the systemic arterial bed [43]. So far, only AEA has been shown to reduce hypoxia-induced vasoconstriction (an important feature of PH), although it failed to modify the vascular caliber under normoxia in murine intra-acinar and pre-acinar arteries [44]. Metabolites of AEA have been demonstrated to mediate hypoxia-induced PH in mice [37]. However, we can exclude this possibility, since 24 h after the final dose of CBD, an increase in lung AEA level and a decrease in RSVP were noticed. The detailed role of other endocannabinoids still remains to be investigated. However, one should keep in mind that the upregulation of the endocannabinoid system under inflammatory conditions is recognized as an autoprotective mechanism in inhibiting disease progression [45].

As we mentioned in the introduction, CBD can increase endocannabinoid levels via FAAH inhibition. Such a mechanism might take place not only in the case of AEA, but also in the case of other FAAH-sensitive *N*-acylethanolamines, i.e., LEA, POEA and EPEA [16]. NAGly is an endogenous FAAH inhibitor [46]. We demonstrated previously that cardiac FAAH activity was inhibited 24 h after the final dose (10 mg/kg) of chronic administration in DOCA-salt and SHR. Interestingly, it was connected with a decrease in cardiac concentrations of 2-AG, DEA and OEA in DOCA-salt and no changes in SHR [16]. Thus, it seems that CBD modifies endocannabinoid levels, dependent on tissue and the model of hypertension.

Fourthly, CBD normalized plasma PAI-1 and t-PA concentrations, which have been found to be strongly enhanced in PH. Similar decreases in plasma t-PA and PAI-1 in rat MCT-induced PH were induced by chronic administration of a phosphodiesterase-5 inhibitor, sildenafil, and a cholesterol lowering drug, simvastatin, which have therapeutic effects in PAH [32].

Fifthly, chronic CBD administration reduced by 60% the leukocyte count that was doubled by MCT, which may result from the well-known anti-inflammatory effects of CBD [47]. They have been described before, e.g., in a murine model of lipopolysaccharide-induced acute lung injury where CBD decreased total lung neutrophil, macrophage and lymphocyte migration into the lungs [17] and potently reduced the inflammatory lung response [48]. However, the potential anti-inflammatory effects of CBD in PH require detailed examination.

The beneficial effects of CBD in PH are not related to its influence on MCT-induced RV hypertrophy and lung edema since CBD did not inhibit the development of the above changes at all. CBD has been suggested to exert beneficial effects in cardiac injury [49] but nobody has so far demonstrated its direct anti-proliferative influence in the heart.

CBD is recognized as a safe drug [50], e.g., in healthy volunteers, oral and/or pulmonary administration of CBD was reported to be safe and well tolerated (600 mg ≈ 8 mg/kg [41]). We have confirmed this finding. Thus, in our hands, chronic CBD did not modify parameters (1) that were not changed by MCT in rats with PH, such as bleeding time, platelet adhesion to collagen and blood parameters other than leukocytes, and (2) of control rats. Only the concentration of NAGly was diminished in the CBD-treated control animals.

### 3.3. Limitations

The current study was limited to the examination of the potential preventive effects of a three-week administration of CBD (10 mg/kg i.p.) on MCT-induced PH in male rats, and more research is needed to generalize our data. Thus, one should keep in mind that other results could be obtained in the case of using (1) female rats, since PAH develops predominantly in woman [27]; (2) other models of experimental PH, e.g., stimulated by hypoxia; (3) therapeutic paradigms of CBD administration; (4) other routes of its administration; (5) a higher dose or a longer period of treatment; and (6) a combination of CBD with other drugs approved for PH therapy (e.g., antagonists of endothelin receptors, bosentan or sildenafil, alone failed to modify lung weight in MCT-induced PH in rats; it was reduced only in response to a combination of both compounds [51])—a drug combination using CBD may be effective in the reduction of cardiac hypertrophy and lung edema. The anti-inflammatory effect of CBD also requires careful examination, especially in lungs.

## 4. Materials and Methods

### 4.1. Animals

All protocols were approved by the Animal Ethics Committee in Olsztyn, Poland (Approval code: no. 88/2018; approval date: 27 November 2018) and were performed in accordance with the European Directive (2010/63/EU). Animal studies were carried out in compliance with the principle of replacement, refinement or reduction. Rats were obtained from the Centre for Experimental Medicine of the Medical University of Bialystok (Białystok, Poland).

Male Wistar rats housed in plastic cages (2 per cage) had free access to water and food pellets and were maintained under a 12 h/12 h light/dark cycle at a constant temperature (22 ±  2 °C) and humidity (50%). The experiments were performed on 56 animals (5–8 weeks old with a baseline body weight of 150–250 g).

### 4.2. Monocrotaline and CBD Treatment

On day 0, MCT was administered as a single, subcutaneous injection (60 mg/kg [24]) in a volume of 3 mL/kg. Age-matched CTR rats received an equal volume of vehicle. Intraperitoneal (i.p.) injections of CBD or its vehicle were performed on day 1 and every 24 h for 21 days. CBD (10 mg/kg) and its vehicle (ethanol, Tween 80, 0.9% NaCl—3:1:16) were prepared immediately before the use and injected in a volume of 1 mL/kg [16]. Rats were assigned randomly to 4 experimental groups—(1) CTR: rats treated with vehicle for MCT and vehicle for CBD (*n* = 14); (2) CTR + CBD: rats treated with CBD and vehicle for MCT (*n* = 14); (3) MCT group: rats treated with MCT and vehicle for CBD (*n* = 14); (4) MCT + CBD group: rats treated with MCT and CBD (*n* = 14). Functional, biochemical and histological examinations were performed 24 h after the final dose of CBD or its vehicle.

### 4.3. Blood Oxygen Saturation Measurements

Blood oxygen saturation was measured using a pulse oximeter (MouseSTAT^®^ Jr. Rodent Pulse Oximeter and Heart Rate Monitor, Kent Scientific Corporation Torrington, CT, USA) attached to the left front paw of the animal.

### 4.4. Hemodynamic Parameters

#### 4.4.1. Measurements of BP

Systolic blood pressure (SBP) and heart rate (HR) were measured in conscious animals by a non-invasive tail-cuff method (Hugo Sachs Elektronik-Harvard Apparatus, March–Hugstetten, Germany). SBP (mmHg) and HR (beats/min) did not differ between groups and were equal in MCT (125 ± 6; 389 ± 15, *n* = 14), MCT + CBD (127 ± 9; 368 ± 23, *n* = 14), CTR (137 ± 4; 366 ± 7, *n* = 14), CTR + CBD (139 ± 5; 361 ± 7, *n* = 14).

#### 4.4.2. Right Ventricular Systolic Pressure Measurements

Rats were anesthetized with pentobarbital sodium (300 µmol/kg, i.p.). A pressure catheter with a sensor for RVSP measurement (SPR-320 Mikro-Tip, Millar, Houston, TX, USA,) was cannulated using the closed chest method through the right jugular vein and placed in the right ventricle [52]. The registration of RVSP waves was recorded on the LabChart 7.3.7 Pro (ADInstruments, Hastings, UK).

### 4.5. Template Bleeding Time

Template BT was measured in anesthetized rats [53]. In brief, a standard incision was made longitudinally on the surface of the tail, starting 2 to 3 cm from the tail root (9 cm from the tip), taking care to avoid the large vessels. Immediately after injury, the tail was inserted into a cylinder with isotonic saline solution at 37 °C. Bleeding time was measured from the moment the tail was surgically cut until bleeding completely stopped (no rebleeding within 30 s).

### 4.6. Platelet Adhesion to Fibrillar Collagen Ex Vivo

After BT measurement, blood samples (5 mL) were drawn and collected from the RV of the heart on anticoagulant (170 mM trisodium citrate, 130 mM citric acid and 101 mM glucose) in a volume ratio of 9:1. The preparation of the washed platelets and their adhesion to fibrillar collagen was performed according to Gromotowicz et al. [53]. In brief, 250 μL washed platelet samples (at final concentration of 3 × 10^5^ platelets/μL) were incubated in an Elvi aggregometer at 37 °C and stirred at 900 rpm with ethylenediaminetetraacetic acid (EDTA) solution (5 mM) to avoid platelet aggregation. After 5 min of pre-incubation, collagen (50 μg/mL) was given, and the platelets were further incubated for 15 min. The platelets were counted optically before and 15 min after adding the collagen in a hemocytometer (Bürker chamber) after dilution with the Unpette system. Index of adhering platelets was calculated using the formula: ((platelet count before adding the collagen—platelet count after adding the collagen)/platelet count before adding the collagen) × 100%.

### 4.7. Hemostatic Parameters and Blood Morphology

Tissue factor, t-PA and PAI-1 plasma levels were determined by enzyme immunoassay (Rat Tissue Factor ELISA Kit, MyBioSource, Inc., San Diego, CA, USA; Rat Active t-PA ELISA Kit and Rat Active PAI-1 ELISA Kit, Innovative Research, Inc., Novi, MI, USA) in a microplate reader (ELx808, BioTek Instruments, Inc., Winooski, VT, USA) according to the manufacturer’s instructions. A blood morphology test was carried out using the hematological analyzer ScilVet ABC Plus+ (HORIBA ABX, Montpellier, France). A volumetric impedance method of counting blood cells was applied [54] for determination of white blood cells (WBCs), red blood cells (RBCs), medium cell volume (MCV), medium content of hemoglobin (MCH), medium cell hemoglobin concentration (MCHC), hemoglobin (HGB), platelet count (PLT), as well as automatic calculation of hematocrit (HCT).

### 4.8. Measurement of Organ Weight

After blood collection, the trachea, lungs and heart were removed. Next, the RV, right atrium and left ventricle plus septum (LV + S) were separated and weighed. The ratio of (RV/LV+S) as an index of RV hypertrophy/Fulton’s index was calculated. The right lung was weighed to estimate the congestion (tissue weight to BW ratio) [25].

### 4.9. Pulmonary Artery Preparation

Rat pulmonary arteries (rPAs) from segmental branches were isolated, carefully cleaned from the lung parenchyma and cut into rings (from the middle portion of each artery; 2 mm in length and ~150 µm internal diameter). The arterial rings were suspended on stainless steel wires (Mulvany-Halpern-type wire myograph, model 620M; Danish Myo Technology, Aarhus, Denmark) in 5-mL organ baths containing Krebs–Henseleit solution with the following composition (in mM) (NaCl 118; KCl 4.8; CaCl_2_ 2.5; MgSO_4_ 1.2; NaHCO_3_ 24; KH_2_PO_4_ 1.2; glucose 11; EDTA 0.03) and were gassed continuously with 95% O_2_ and 5% CO_2_ at 37 °C (pH 7.4). Rat vessels were set up at tensions equivalent to their mean in vivo right ventricular pressure using the Laplace equation [9]. The rPA rings were allowed to equilibrate for 30 min. Tensions were measured and recorded on the LabChart 7.3.7 Pro (ADInstruments, Hastings, UK). After the equilibration period, all of the rings were exposed to two stimuli of KCl (50 mM) to establish tissue viability. After washing with KCl, the integrity of the vessel endothelium was checked for submaximal preconstriction with phenylephrine (10 µM), followed by the induction of at least 50% relaxation in response to acetylcholine (1 µM, [13]).

#### Functional Studies of rPAs

In each individual preparation, only one CRC was determined. All experiments were performed in paired vessels. The vasodilatory effects of Ach (0.001–300 μM) or SNP (0.0001–30 μM) were examined in rPAs pre-contracted with U46619 (0.1–0.3 µM; a concentration approximately equivalent to its EC_60_). In all rPA experimental groups, U46619-induced increases in tone were comparable (in mN: CTR, 1.9 ± 0.3, *n* = 15; CTR+CBD, 1.9 ± 0.2, *n* = 15; MCT, 2.1 ± 0.3, *n* = 15; MCT+CBD, 2.1 ± 0.2, *n* = 15, respectively).

To examine vascular contractile functions, isolated rPAs were exposed to increasing concentrations of U46619 (0.0001–3 µM) or phenylephrine (0.001–30 µM). There was comparable contractile arterial tone in all of the examined rPAs, as we did not observe any differences in response to KCl (50 mM in rPAs) given prior to each experiment (CTR, 3.4 ± 0.4, *n* = 15; CTR+CBD, 3.4 ± 0.2, *n* = 15; MCT, 3.6 ± 0.4, *n* = 15; MCT+CBD, 3.3 ± 0.4, *n* = 15).

### 4.10. Morphometric Analysis of rPAs

Morphometric analysis was conducted on 32 rats, i.e., 8 rats from each experimental group. Three sections (from the superior, middle and interior) of the right lung were taken from each rat and, after fixing in 10% buffered formalin, were routinely embedded in paraffin. The paraffin blocks of tissue were cut into 4-μm-thick sections (three sections from each lung fragment) of each animal and stained with hematoxylin and eosin for microscopic evaluation and documentation [using an Olympus BX41 microscope with an Olympus DP12 camera (Olympus Corporation, Tokyo, Japan)].

Stained sections were used to identify, evaluate and measure the medial wall thickness of the PAs. Morphometric analysis was performed using NIS-Elements Advanced Research software from Nikon. The ratio of percentage vascular wall thickness (WT.%) was measured in 10 arteries (ranging in external diameter from 40 to 100 µm) per lung section from each rat. For each artery WT.% was calculated as follows: 100% × (outer diameter – internal diameter)/outer diameter) [24].

### 4.11. Immunohistochemistry

For immunohistochemical analysis, the EnVision method was used according to Kloza et al. [55], using antibodies against von Willebrand factor (vWF) (1:2000, 2 h incubation at room temperature (RT), Polyclonal Rabbit Anti-Human (no cat. A 0082); DakoCytomation, Glostrup, Denmark). Antigen retrieval was performed before commencing immunohistochemical staining for vWF using Target Retrieval Solution (S1699; Dako, Glostrup, Denmark). A negative control was included, in which the antibody was replaced by normal rabbit serum (Vector Laboratories, Burlingame, CA, USA) at the respective dilution (no staining), and a positive control was included using rat lung stained for vWF. Immunohistochemical staining was evaluated on an Olympus BX41 microscope with an Olympus DP12 camera under 200× magnification.

### 4.12. Quantification of Endocannabinoids and Endocannabinoid-Related Lipids

The lung was perfused with 0.9% saline, snap-frozen with liquid nitrogen and stored at −80 °C. Next, the lung was pulverized in liquid nitrogen to examine levels of endocannabinoids. *N*-arachidonoylethanolamine (AEA), 2-Arachidonoylglycerol (2-AG), almitoylethanolamide (PEA), oleoylethanolamide (OEA), stearoyl ethanolamide (SEA), palmitoleoylethanolamide (POEA), linolenoylethanolamide (LEA), arachidonoylglycine (NAGly), docosahexaenoyl ethanolamide (DHEA), docosatetraenoyl ethanolamide (DEA), homo-γ-linolenyl ethanolamide (HEA), 2-linoleoylglycerol (2-LG) and eicosapentaenoylethanolamide (EPEA) were quantified using ultra-high performance liquid chromatography tandem mass-spectrometry (LCMS 8060, Shimadzu, Kioto, Japan) [56]. A Poroshell 120 EC-C18 column (3.0 mm × 150 mm, 2.7-micron) was employed. The initial chromatographic conditions were 70% acetonitrile (ACN) in water containing 0.1% (*v*/*v*) of formic acid as an ionizing agent. After isocratic development for 1 min, a gradient was applied up to 80% ACN from 1–5 min, followed by a second gradient up to 88% ACN from 5–15 min; then, 100% ACN was reached after 0.5 min. These conditions were kept constant until the end of the chromatographic step that finished at 25 min. The temperature of the chromatographic column and the flow rate were maintained at 15 °C and 800 µL/min, respectively. Endocannabinoids were extracted using solid phase extraction (SPE) and analyzed in positive-ion mode (MRM). AEA-d_8,_ 2-AG-d_8,_ and OEA-d_4_ were used as internal standards for quantification. Transitions of the precursors to the product ions were as follows: *m*/*z* 348.3→62.15 for AEA, *m/z* 379.3→287.25 for 2-AG, m/z 324.3→62.0 for LEA, *m/z* 355.0→263.0 for 2-LG, 326.3→62.0 for OEA, *m/z* 298.3→62.0 for POEA, *m/z* 372.3→62.0 for DHEA, *m/z* 300.3→62.0 for PEA, *m/z* 376.3→62.0 for DEA, *m/z* 314.5→62.0 for HEA, *m/z* 328.3→62.0 for SEA, *m/z* 346.3→62.0 for EPEA, *m/z* 362.10→287.25 for NAGLy, *m/z* 356.2→63.05 for AEA-d8, *m/z* 387.3→294.0 for 2-AG-d8 and *m/z* 330.20→66.15 for OEA-d4.

### 4.13. Drugs

(-)-Cannabidiol (CBD; THC-1073G-1) was obtained from THC Pharm, Frankfurt, Germany; ethanol (BA6420113) and natrium chloride (NaCl; BA4121116) were obtained from POCH, Gliwice, Poland; Crotaline (MCT; C2401-1G) and Tween 80 (P1754) were obtained from Sigma-Aldrich, Munich, Germany. MCT was dissolved in 1 M HCl, and the pH was adjusted to 7.4 with 1 M NaOH; pentobarbital sodium was acquired from Biowet, Puławy, Poland. Acetylcholine chloride (A6625; Sigma Chemical, St. Louis, MO, USA), (R)-l-phenylephrine hydrochloride (P6126; Sigma-Aldrich, Steinheim, Germany) and sodium nitroprusside dehydrate (71778; Sigma-Aldrich, Steinheim, Germany) were dissolved in deionized water. A stock solution of U46619 ((5Z,8Z,11Z,14Z)-5,8,11,14-eicosatetraenoic acid; Tocris Bioscience, Bristol, UK) was dissolved in ethanol (0.1% *v/v*) 0.01 µM and its final concentrations were prepared by dilutions with deionized water. Collagen (Chronolog, Havertown, PA, USA); citric acid, EDTA, glucose, glycine, magnesium chloride, sodium chloride, potassium chloride, sodium bicarbonate and trisodium citrate were provided by Polish Chemical Reagents (Poland). Anti-rat TF (Rat TF ELISA Kit, MBS161467, MyBiosource San Diego, CA, USA), active rat tPA and a PAI-1 ELISA Kit (RTPAKT-1KIT, RPAIKT-1KIT, Molecular Innovations; respectively) were used in this study. Polyclonal Rabbit Anti-Human Von Willebrand Factor (no cat. A 0082) was acquired from DakoCytomation, Glostrup, Denmark. Reagents for routine histological hematoxylin and eosin staining and a secondary antibody EnVision+ Kit (horseradish peroxidase, rabbit) were obtained from Dako Denmark (Glostrup, Denmark).

### 4.14. Statistical Analysis

All results are expressed as mean ± SEM of *n* animals. Contractile responses to U46619 and phenylephrine are presented as a percentage of the reference contraction response to 50 mM of KCl after the equilibration period at the beginning of each experiment. The vasodilatory effects of acetylcholine and sodium nitroprusside are expressed as a percentage of relaxation of the isometric submaximal contraction induced by U46619 (0.1–0.3 μM). GraphPad Prism 5.0 software (La Jolla, CA, USA) was used to plot the mean data as sigmoidal CRCs. The curves were then used to determine the potency (pEC_50_, the negative logarithm of the concentration causing the half-maximum effect) and the maximum effect (E_max_) values as the effects of the highest agonist concentration. The rightward shifts of CRCs relative to the control curve were calculated on the basis of the EC_50_ values. EC_50_ values were transformed into pEC_50_ values (the negative logarithms of the EC_50_ values). Intergroup statistical comparisons were carried out using a one-way analysis of variance (ANOVA), followed by the Bonferroni multiple comparison test. Post hoc tests were run only if F achieved the necessary level of statistical significance and there was no significant variance inhomogeneity. Differences were considered to be statistically significant if *p* < 0.05.

## 5. Conclusions

Our results reveal for the first time that chronic CBD administration strongly ameliorated MCT-induced PH (in the current study) but did not diminish systemic BP in experimental primary and secondary hypertension [16]. Importantly, CBD stimulated a pronounced decrease in RVSP (which is enhanced in PH) and improved the blood oxygen saturation, which was decreased in the MCT-treated rats. The beneficial effects of CBD appear to be related mainly to the reduction of pulmonary vascular resistance, which could result from the improvement of endothelial efficiency and function and the normalization of hemostatic alterations. The CBD-stimulated improvement of blood leukocyte count and an increase lung endocannabinoid levels may be of additional benefit in PAH treatment. Importantly, we did not observe any side effects of CBD both in rats with PH and controls. In summary, our preclinical studies indicate that CBD may be a useful therapeutic option in PAH. Unfortunately, CBD did not diminish the RV hypertrophy and lung edema in PH, but one therapeutic approach may involve CBD as an adjuvant therapy in combination with currently available therapies for PAH. Moreover, the pulmonary-protective and anti-inflammatory properties of CBD may be a beneficial and safe addition to the therapeutic armamentarium against other respiratory diseases. However, further detailed pre- and clinical studies are required in order to confirm our promising observations.

## Figures and Tables

**Table 1:** Influence of chronic administration of cannabidiol on the vasorelaxant effects of acetylcholine (Ach) and sodium nitroprusside (SNP) and vasoconstriction effects of phenylephrine (PHE) and thromboxane A2 analogue (U46619) in the pulmonary arteries of control (CTR) and monocrotaline (MCT)-induced pulmonary hypertension.

Group	CTR	CTR+CBD	MCT	MCT+CBD
Ach	(7)	(7)	(7)	(7)
*pEC_50_*	6.0 ± 0.1	6.0 ± 0.1	5.3 ± 0.1 ***	5.4 ± 0.1 **
*E_max_ (%)*	72.7 ± 2.9	69.0 ± 7.4	43.1 ± 3.9 *	77.3 ± 8.0 ^##^
SNP	(8)	(8)	(8)	(8)
*pEC_50_*	6.9 ± 0.1	7.0 ± 0.1	6.2 ± 0.1 ***	6.7 ± 0.1 ^##^
*E_max_ (%)*	90.8 ± 6	90.9 ± 7.0	70.8 ± 3.9 *	101.4 ± 5.9 ^##^
PHE	(7)	(7)	(7)	(7)
*pEC_50_*	7.1 ± 0.2	7.0 ± 0.1	6.8 ± 0.1	6.8 ± 0.1
*E_max_ (%)*	40.5 ± 9.3	41.8 ± 5.1	71.4 ± 7.0	53.4 ± 3.6
U46619	(8)	(8)	(8)	(8)
*pEC_50_*	7.0 ± 0.1	6.9 ± 0.1	7.2 ± 0.1	6.9 ± 0.1
*E_max_ (%)*	85.6 ± 7.1	98.5 ± 9.0	118.6 ± 7.8 *	85.5 ± 6.9 ^#^

Cannabidiol (CBD) 10 mg/kg or its vehicle was injected intraperitoneally every 24 h for 21 days. Data are expressed as mean ± SEM. The number of animals is shown in parentheses. Vasodilator and contractile responses are shown as a percentage of the reference response of the isometric contraction induced by U46619 (0.1 µM) or 50 mM KCl, respectively. *^,#^ *p* < 0.05; **^.##^ *p* < 0.01; *** *p* < 0.001 compared to the respective control groups (* CTR or ^#^ MCT), as determined by one-way ANOVA, followed by Bonferroni’s multiple comparison test.

**Table 2:** Influence of chronic administration of cannabidiol on blood morphology, bleeding time and platelet adhesion to collagen in plasma from control (CTR) and monocrotaline (MCT)-induced pulmonary hypertension.

Parameter	*n*	CTR	CTR+CBD	MCT	MCT+CBD
WBC (10^3^/µL)	8	2.4 ± 0.2	2.5 ± 0.4	5.0 ± 0.4 ***	3.6 ± 0.3 ^#^
RBC (10^6^/µL)	8	6.9 ± 0.2	6.9 ± 0.2	7.2 ± 0.2	7.4 ± 0.3
HCT (%)	8	40.5 ± 0.7	40.7 ± 1.2	42.1 ± 1.3	44.1 ± 2.1
HGB (g/dL)	8	13.6 ± 0.2	13.7 ± 0.2	14.5 ± 0.3	15.2 ± 0.6
MCV (fl)	8	58.0 ± 0.7	59.0 ± 0.3	57.7 ± 0.6	60.2 ± 0.8
MCH (pg/cell)	8	19.6 ± 0.3	19.9 ± 0.3	19.6 ± 0.4	20.5 ± 0.4
MCHC (g/dL)	8	33.7 ± 0.2	33.8 ± 0.3	34.3 ± 0.4	34.0 ± 0.3
PLT (10^3^/µL)	8	575.8 ± 20.1	620.0 ± 7.8	611.9 ± 37.4	554.4 ± 22.4
Bleeding time (s)	8	97.6 ± 4.9	104.3 ± 4.0	101.7 ± 5.2	107.3 ± 9.5
Platelet adhesion to collagen (%)	8	29.3 ± 1.5	29.2 ± 2.1	32.6 ± 2.2	30.5 ± 1.7

HCT: hematocrit; HGB: hemoglobin; MCV: medium cell volume, MCH: medium content of hemoglobin, MCHC: medium cell hemoglobin concentration, PLT: platelet count; RBC: red blood cells, WBC: white blood cells. Cannabidiol (CBD) 10 mg/kg or its vehicle was injected intraperitoneally every 24 h for 21 days. Data are presented as means ± SEM; ^#^ *p* < 0.05; *** *p* < 0.001, compared to the respective group (* CTR, ^#^ MCT); as determined by one-way ANOVA, followed by Bonferroni’s multiple comparison test.

## Abbreviations

ACN	acetonitrile
AEA	anandamide
2-AG	2-arachidonoylglycerol
ANOVA	analysis of variance
BP	blood pressure
BT	bleeding time
BW	body weight
CBD	cannabidiol
CRC	concentration response curve
CTR	control
DEA	docosatetraenoyl ethanolamide
DHEA	docosahexaenoyl ethanolamide
EPEA	eicosapentaenoyl ethanolamide
FAAH	fatty acid amide hydrolase
HEA	homo-γ-linolenyl ethanolamide
HR	heart rate
LEA	linolenoyl ethanolamide
2-LG	linoleoylglycerol
LV	left ventricle
MCT	monocrotaline
NAGly	*N*-arachidonoyl glycine
OEA	oleoyl ethanolamide
PAH	pulmonary arterial hypertension
PH	pulmonary hypertension
PAI-1	plasminogen activator inhibitor type 1
PEA	palmitoyl ethanolamide
POEA	palmitoleoyl ethanolamide
rPAs	rat pulmonary arteries
RVSP	right ventricular systolic pressure
RV	right ventricle
SBP	systolic blood pressure
SEA	stearoyl ethanolamide
TF	tissue factor
t-PA	tissue plasminogen activator
vWF	von Willebrand factor
